# Enhancing Nitrification at Low Temperature with Zeolite in a Mining Operations Retention Pond

**DOI:** 10.3389/fmicb.2012.00271

**Published:** 2012-07-30

**Authors:** Misha Miazga-Rodriguez, Sukkyun Han, Brian Yakiwchuk, Kai Wei, Colleen English, Steven Bourn, Seth Bohnert, Lisa Y. Stein

**Affiliations:** ^1^Department of Biological Sciences, University of AlbertaEdmonton, AB, Canada; ^2^Environmental Monitoring, Diavik Diamond Mines Inc.Yellowknife, NT, Canada

**Keywords:** nitrification, ammonia-oxidizing bacteria, cold environments, mining, zeolite, biofilm

## Abstract

Ammonium nitrate explosives are used in mining operations at Diavik Diamond Mines Inc. in the Northwest Territories, Canada. Residual nitrogen is washed into the mine pit and piped to a nearby retention pond where its removal is accomplished by microbial activity prior to a final water treatment step and release into the sub-Arctic lake, Lac de Gras. Microbial removal of ammonium in the retention pond is rapid during the brief ice-free summer, but often slows under ice cover that persists up to 9 months of the year. The aluminosilicate mineral zeolite was tested as an additive to retention pond water to increase rates of ammonium removal at 4°C. Water samples were collected across the length of the retention pond monthly over a year. The structure of the microbial community (bacteria, archaea, and eukarya), as determined by denaturing gradient gel electrophoresis of PCR-amplified small subunit ribosomal RNA genes, was more stable during cold months than during July–September, when there was a marked phytoplankton bloom. Of the ammonia-oxidizing community, only bacterial *amoA* genes were consistently detected. Zeolite (10 g) was added to retention pond water (100 mL) amended with 5 mM ammonium and incubated at 12°C to encourage development of a nitrifying biofilm. The biofilm community was composed of different *amoA* phylotypes from those identified in gene clone libraries of native water samples. Zeolite biofilm was added to fresh water samples collected at different times of the year, resulting in a significant increase in laboratory measurements of potential nitrification activity at 4°C. A significant positive correlation between the amount of zeolite biofilm and potential nitrification activity was observed; rates were unaffected in incubations containing 1–20 mM ammonium. Addition of zeolite to retention ponds in cold environments could effectively increase nitrification rates year-round by concentrating active nitrifying biomass.

## Introduction

Diavik Diamond Mine Inc. (DDMI), located within Lac de Gras of the Northwest Territories, Canada, utilizes ammonium nitrate as an explosive for mining operations. A by-product of incomplete ammonium nitrate combustion is residual ammonium $\left({\text{NH}}_{\text{4}}^{\text{ + }}\right)$ that is piped from the mine pit into a retention pond where it is oxidized to nitrate $\left({\text{NO}}_{\text{3}}^{-}\right)$ via nitrification. ${\text{NO}}_{\text{3}}^{-}$ is then removed from retention pond water by a wastewater treatment plant located on site before release back into Lac de Gras. The use of a retention pond is generally common for remediating residual nitrogenous wastes from mining operations, though in cold environments, nitrogen removal can be slowed by temperature, substrate availability, and salinity effects on microbial populations (Hwang and Oleszkiewicz, 2007; Zaitsev et al., 2008; Ducey et al., 2010; Karkman et al., 2011; Rodriguez-Caballero et al., 2012). In addition, cold temperatures can result in decreased diversity and richness of nitrifying communities (Urakawa et al., 2008; Karkman et al., 2011) or shifts in nitrifying community composition (Rodriguez-Caballero et al., 2012), which can influence the rate at which nitrogenous substrates are metabolized.

The first step of nitrification is the biological oxidation of ammonia (NH_3_) to nitrite $\left({\text{NO}}_{\text{2}}^{-}\right)$ as mediated by ammonia-oxidizing bacteria (AOB) and ammonia-oxidizing archaea (AOA). ${\text{NO}}_{\text{2}}^{-}$ is further oxidized to ${\text{NO}}_{\text{3}}^{-}$ by nitrite-oxidizing bacteria to complete the nitrification process (Ward, 2011). At DDMI, the oxidation of NH_3_ in the retention pond proceeds readily during the warm summer months, whereas in cold winter months, nitrification tends to slow. The slowing of nitrification results in transient accumulation of ${\text{NH}}_{\text{4}}^{\text{ + }}$ beyond the maximum level established by mining operating procedures, which can then slow the rate at which water can be treated for ${\text{NO}}_{\text{3}}^{-}$ removal and released back into Lac de Gras, ultimately leading to a slowdown of mining operations.

An effective method to speed nitrification rates at low temperatures in other wastewater treatment and mining systems is promotion of nitrifying biofilms that promote growth and retention of microbial biomass (Andreottola et al., 2000; Choi et al., 2008; Zaitsev et al., 2008). Collectively, these prior studies demonstrated the effectiveness of fixed-bed, mobile bed, and aerated submerged biofilm reactors in the removal of ${\text{NH}}_{\text{4}}^{\text{ + }}$ down to 4–5°C and also showed resilience of developed nitrifying biofilms to temperature fluctuation. Another method for speeding nitrification at low temperature is to physically heat nitrifying biofilm supports; however, a test of this approach yielded inconsistent results and was impractical in terms of cost, energy, and infrastructure requirements (Gebert and Wilderer, 2000). A third approach involved bioaugmentation of bioreactors with nitrifying biomass, which effectively maintained nitrification activity after a cold shock from 20 to 10°C (Head and Oleszkiewicz, 2004). This approach required constant application of biomass to avoid a rapid decline in nitrification rates, which is impractical for facilities that lack capacity for maintaining stocks of viable nitrifying microorganisms.

While each of these methods are effective at enhancing nitrification rates at low temperatures, they are impractical for the remote area and harsh climate at DDMI and other mining and wastewater operations located in the far north. The above methods also require relatively extensive infrastructure and technical support. Therefore, the present study tested the hypothesis that addition of zeolite to retention pond water samples promotes development of native nitrifying biofilms that remain active at 4°C. If successfully demonstrated, application of zeolite could offer an inexpensive solution to speeding remediation of nitrogenous wastes in any cold aquatic wastewater or mining ecosystem.

Zeolite is a high surface area, microporous, aluminum silicate mineral with cation exchange properties. Zeolite amendment has been shown to effectively enhance nitrification rates in conventional waste water treatment systems by increasing and localizing nitrifying biomass due to its ability to adsorb ammonium and provide surface area for microbial attachment and growth (Furukawa et al., 2000; Son et al., 2000; Pak et al., 2002). Addition of zeolite requires no additional infrastructure or technical support and can remain active in the environment until its physical removal. After examining the seasonal dynamics of the native microbial population residing in the DDMI retention pond, with particular attention to the ammonia-oxidizing microbial population, we determined whether addition of zeolite to water samples could encourage formation of a nitrifying biofilm that could then be used to enhance the rate of ${\text{NH}}_{\text{4}}^{\text{ + }}$ removal at 4°C, the average annual temperature of the DDMI retention pond. This study offers a proof-of-principle for the application of zeolite to cold aquatic ecosystems to speed removal of nitrogenous wastes.

## Materials and Methods

### Sample collection and processing

**Figure 1:**
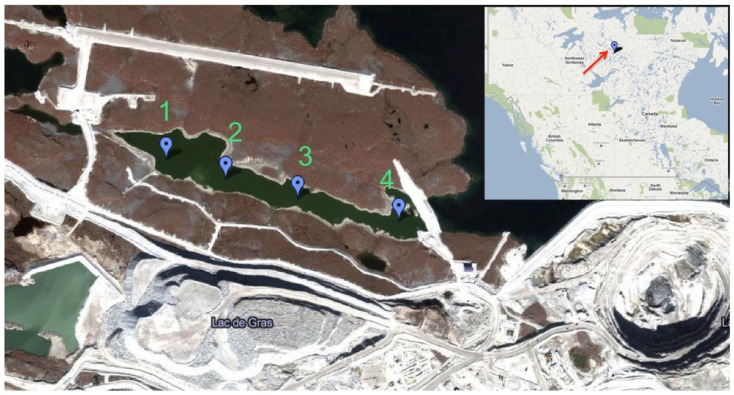
**Map of retention pond and location of Diavik Diamond Mines Inc. (courtesy of DDMI) in the Northwest Territories, Canada**. Water sampling sites 1–4 are indicated by balloons. Only sites 1 and 3 were accessible during ice cover (September 2009–May 2010).

The retention pond at DDMI accepts ca 15,000 m^3^ of water per day containing an average of 7 mg L^−1^ ${\text{NH}}_{\text{4}}^{\text{ + }}$ as dependent on mining activity. Water samples (ca. 20 L) were collected from sites 1 and 3 in April 2009 and from September 2009 to May 2010 via auger holes through the ice cover (Figure 1). From June to August 2009, water was collected from near the surface at sites 1–4 by bucket. Dissolved oxygen (DO), pH, temperature, turbidity, and conductivity were measured at the site of sample collection using a HydroLab (Hach Hydromet, Loveland, CO, USA). ${\text{NH}}_{\text{4}}^{\text{ + }}$ was measured using the automated phenate method (Clesceri et al., 1998). Water samples were poured into ultraclean polyethylene bags, sealed, and shipped to the University of Alberta within 48 h of collection; shipping temperature did not exceed 12°C and freezing was avoided.

### Potential nitrification activity

Immediately upon arrival at the University of Alberta, duplicate water samples (100 mL) from each sample site (denoted in Table 2 footnote) were added to 125 mL Erlenmeyer flasks and amended with 1 mM NH_4_Cl. Flasks were incubated statically at 4 and 12°C for 15–30 days. ${\text{NO}}_{\text{3}}^{-}$ was measured via colorimetric assay every 2–3 days using the NitraVer 5 kit (Hach, Loveland, CO, USA) according to manufacturer instructions. Background ${\text{NO}}_{\text{3}}^{-}$ levels measured in duplicate autoclaved water samples, also amended with 1 mM NH_4_Cl, were subtracted from each measurement. Final ${\text{NO}}_{\text{3}}^{-}$ concentrations were determined by comparison to a standard curve of KNO_3_ from 0.2 to 1.5 mM. Potential nitrification activity (PNA) rates were determined by integrating duplicate ${\text{NO}}_{\text{3}}^{-}$ measurements made on duplicate samples for water collected at each site as denoted in the footnote for Table 2.

### Development of biofilm on zeolite

Zeolite (RENA, Chalfont, PA, USA) was washed with distilled water, and autoclaved. Sterile zeolite (10 g) was added to retention pond water samples (100 mL) collected from site 3 (Figure 1) in January 2010 and May 2010 amended with 1 mM NH_4_Cl. Samples were incubated at 12°C for 1 month, at which point the ${\text{NH}}_{\text{4}}^{\text{ + }}$ had been completely converted ${\text{NO}}_{\text{3}}^{-}$ (i.e., over 1 mM ${\text{NO}}_{\text{3}}^{-}$ was measured in the overlaying water, accounting for oxidation of the 1 mM NH_4_Cl amendment plus the ${\text{NH}}_{\text{4}}^{\text{ + }}$ in the native water sample) indicating the presence of active nitrifying microorganisms. The developed zeolite biofilm was collected by filtration and added back to fresh water samples at 10 g per 100 mL, or indicated quantities. Quadruplicate samples were amended with NH_4_Cl at 5 mM to test different sampling dates and the effect of increasing amounts of zeolite biofilm, or at indicated concentrations to test effects of ${\text{NH}}_{\text{4}}^{\text{ + }}$ on rates of PNA. Incubations containing sterile, rather than pre-incubated, zeolite were prepared as a control. Samples were incubated at 4°C and ${\text{NO}}_{\text{3}}^{-}$ was measured over time such that rates of PNA with zeolite biofilm could be compared to water samples amended with sterile zeolite and without zeolite. ${\text{NO}}_{\text{2}}^{-}$, measured using a standard colorimetric assay (Clesceri et al., 1998), was below the level of detection in all of the samples.

### Microbial community characterization

Water samples (ca. 1 L) were filtered through 0.22 μm filters to collect microbial biomass, which were then stored in 1 mL sucrose lysis buffer (20 mM Na-EDTA, 400 mM NaCl, 0.75 M sucrose, 50 mM Tris-Cl pH 9.0) at −80°C prior to processing. DNA was extracted from the filters as described elsewhere (Giovannoni et al., 1996). Zeolite bioflims were crushed using a sterilized mortar and pestle. Nucleic acids were extracted from 500 mg crushed material using the FastDNA kit for soil according to manufacturer protocols (MP Biomedicals, Solon, OH, USA), modified by addition of 20 mg skim milk protein.

Amplification of partial bacterial, archaeal, and eukaryotic small subunit ribosomal RNA (SSU rRNA) genes for denaturing gradient gel electrophoresis (DGGE) was achieved using universal primer sets (Table 1). PCR amplification (50 μl reactions) contained ca. 50 ng template DNA, 10 mM Tris-Cl (pH 8.5), 40 mM KCl, 100 μM each dNTP, 1.5 mM MgCl_2_, 2 U Taq polymerase, and 0.2 μM of each primer. Following initial denaturation at 94°C (5 min), 30 cycles were run at 30 s denaturation at 94°C, 30 s at 55°C (47°C for bacterial *amoA* primers), and 30 s extension at 72°C, with a final extension at 72°C. DGGE was performed on triplicate sub-samples from each sampling date and from every sampled location using the D-code universal mutation detection system (BioRad Laboratories, Inc., Hercules, CA, USA) as described in detail previously (Kulp et al., 2006). Gradient gels ranged from 40 to 60% denaturant; 100% denaturant contained 7 M urea and 40% formamide in 8% (w/v) in polyacrylamide gels (Muyzer et al., 1993). Marker lanes containing a mixture of 12 known PCR products were used to normalize band migration distances among multiple gels. Dendrograms comparing similarities in banding patterns from sample to sample were constructed using GelCompar II (version 4.0; Applied Maths, Kortrijk, Belgium) using the unweighted pair group method with arithmetic means based on Dice correlation coefficients.

**Table 1:** **PCR primers used in this study**.

Primer set	Target	Sequence (5′–3′)	Amplicon length (bp)	Reference
341F*	Bact 16S	CCTACGGGAGGCAGCAG	177	Muyzer et al. (1993)
518R	rRNA	ATTACCGCGGCTGCTGG	
pArch340F*	Arch 16S	TACGGGGYGCASCAG	175	Øvreås et al. (1997)
pArch519R	rRNA	TTACCGCGGCKGCTG	
Euk1A	Euk 18S	CTGGTTGATCCTGCCAG	559	Díez et al. (2001)
Euk516R*	rRNA	ACCAGACTTGCCCTCC
amoAf-i*	Bact *amoA*	GGGGITTITACTGGTGGT	491	Hornek et al. (2006)
amoAr-i		CCCCTCIGIAAAICCTTCTTC
Arch-amoAf	Arch *amoA*	STAATGGTCTGGCTTAGACG	635	Francis et al. (2005)
Arch-amoAr*		GCGGCCATCCATCTGTATGT

*GC clamp (40 bp) added for DGGE-PR.

Ammonia monooxygenase (*amoA*) genes were PCR-amplified from extracted nucleic acids using published primer sets (Table 1). Amplification products were cloned into TOPO vectors, and positive inserts were bi-directionally sequenced using Big Dye and Sanger Sequencing according to manufacturer instructions (Life Technologies, Carlsbad, CA, USA). Sequences for *amoA* genes can be found in GenBank under accession numbers JX173792-97. Quantitative PCR was performed on triplicate water samples from each month using the bacterial 16S rRNA and *amoA* primer sets, but without GC clamps (Table 1). qPCR reactions (30 μL) had the same proportion of reagents as described above for DGGE-PCR, but also contained 1x SYBR Green I (Molecular Probes, Eugene, OR, USA). Running conditions on an ABI StepOne Plus (Life Technologies, Carlsbad, CA, USA) were 95°C for 5 min followed by 45 cycles at 94°C (10 s), 55 or 47°C (20 s), and 85°C (10 s). *C*_t_ values were calculated from standard curves of cloned 16S rRNA or *amoA* genes from *Nitrosomonas europaea* (ATCC 19718) diluted from 10^2^ to 10^8^ copies per reaction. Triplicate reactions were analyzed for each samples to calculate standard deviations.

## Results

### General microbial community

**Figure 2:**
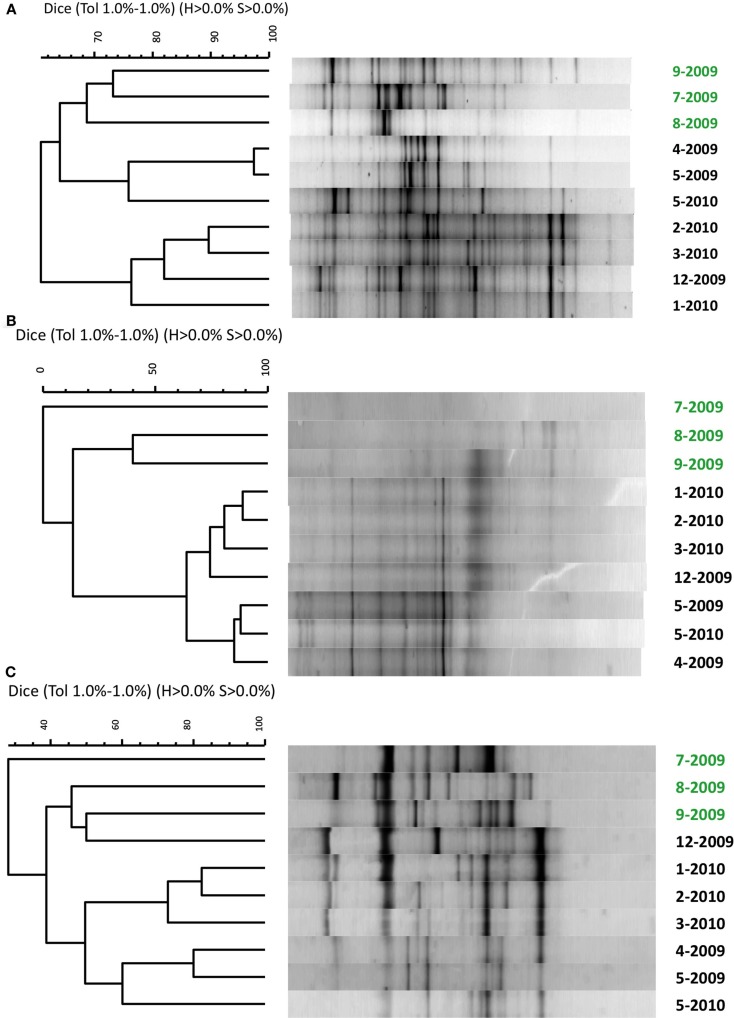
**Similarity comparison of denaturing gradient gel electrophoresis profiles of PCR-amplified small subunit ribosomal RNA genes for (A) bacteria, (B) archaea, and (C) eukarya**. Because DGGE profiles were identical from at each site across the retention pond and from each replication (data not shown), only representative samples from site 3 from each month are shown. Water samples were collected from April 2009 to May 2010 with month (number) and year indicated on the right axis. Samples in green font indicate summer months with phytoplankton bloom.

The broad microbial community within the DDMI retention pond, as assessed by DGGE banding patterns of PCR-amplified SSU rRNA genes, was more similar over the cold months, based on Dice correlation coefficients, than during the warmest months (July–September) over 1 year of monitoring (Figure 2). A phytoplankton bloom was evident over the warm months from the bright green color of the water samples. Based on qPCR of 16S rRNA genes, the bacterial population was significantly more abundant in August and September than in other months (Table 2). No significant variability in community composition was observed from samples collected across the retention pond for any month, indicating homogenous distribution of the microbial community within this environment (data not shown).

**Table 2:** **Relative abundances of bacterial *amoA* genes and potential nitrification activity in the retention pond water by month**.

Sample date	# Bacterial *amoA* gene copies (×10^7^)^a^	# Bacterial 16S rRNA gene copies (×10^7^)^a^	Potential nitrification activity at 12°C^b^
July 2009	0.75 (0.02)	3.89 (0.08)	1.09 (0.09)
August 2009	0.49 (0.006)	6.44 (0.20)	ND^c^
September 2009	0.12 (0.01)	7.82 (1.29)	3.11 (0.10)
December 2009	0.41 (0.002)	4.90 (0.39)	0.80 (0.03)
January 2010	0.41 (0.002)	5.90 (0.02)	0.85 (0.03)
February 2010	0.10 (0.02)	2.27 (0.16)	1.67 (0.06)
March 2010	0.02 (0.002)	2.82 (0.19)	ND
May 2010	0.03 (0.004)	4.02 (0.19)	1.62 (0.68)

*^a^Number of gene copies detected by qPCR per mL water sample*.

*^b^Samples from sites (shown in Figure 1) used for PNA measurements were: 1–4 for July and August 2009, and 1 and 3 for September and December 2009, January–May 2010. Measurements were made in duplicate for each sample from each site, and rate measurements were combined from all samples and all sites for each month to determine standard deviations*.

*^c^ND, not determined*.

### Ammonia-oxidizing microbial community

Bacterial *amoA* genes (encoding the alpha subunit of ammonia monooxygenase) were detected by PCR in all water samples, whereas two archaeal *amoA* amplicons (593/596 and 591/594 nucleotide match to uncultivated members of group I.1a marine crenarchaeota) were only detectable in the March 2010 sample using nested PCR (data not shown). The numbers of bacterial *amoA* genes as determined by qPCR were highest in the July 2009 sample, and lowest in the March 2010 and May 2010 samples (Table 2). Total abundance of bacterial 16S rRNA genes did not correlate with bacterial *amoA* gene abundance, and neither gene abundance nor the ratio of *amoA*/SSU rRNA gene copy number correlated with laboratory measurements of PNA. Thus, correlations of *amoA* gene abundance or PNA rates with physicochemical parameters of the retention pond water were examined (Table 3). Positive correlations between pH (Pearson correlation coefficient = 0.69; *P* < 0.05) and DO (Pearson correlation coefficient = 0.68; *P* < 0.05) of water samples with bacterial *amoA* gene abundance were found, whereas a negative correlation was found between PNA rate and water ${\text{NH}}_{\text{4}}^{\text{ + }}$ concentration (Pearson correlation coefficient = −0.68; *P* < 0.05).

**Table 3:** **Physicochemical parameters of retention pond water**.

Sample date	Temp. (°C)	pH	DO (mg/L)	Conductivity (μS/cm)	Turbidity (NTU)	Ammonia (mg/L)^a^
April 09	1.86 (0.15)	8.55 (0.07)	10.15 (0.22)	569 (9)	26.2 (19.1)	1.67 (0.05)
May 09	1.93 (0.18)	8.68 (0.15)	10.21 (0.26)	582 (8)	2.62 (1.12)	1.68 (0.16)
July 09	7.02 (1.71)	9.60 (0.42)	16.26 (3.07)	397 (16)	4.22 (2.79)	ND
August 09	10.45 (1.77)	9.62 (0.60)	11.92 (3.62)	550 (35)	1.38 (2.03)	0.45 (0.78)
September 09	5.45 (0.15)	9.51 (0.06)	12.51 (0.19)	571 (10)	0.21 (0.19)	BDL
December 09	1.47 (0.044)	8.24 (0.03)	10.42 (0.69)	614 (125)	12.86 (8.09)	0.22 (SM)
January 10	2.13 (0.71)	7.83 (0.25)	9.22 (2.05)	506 (134)	9.03 (8.79)	0.62 (0.26)
February 10	2.17 (0.50)	7.85 (0.28)	9.04 (3.08)	513 (157)	15.83 (7.12)	0.50 (0.04)
March 10	3.40 (SM)	7.86 (0.22)	11.95 (0.35)	403 (11)	7.96 (5.58)	ND
April 10	5.70 (2.76)	7.79 (0.05)	11.80 (SM)	396 (SM)	4.12 (4.00)	ND
May 10	5.00 (0.49)	8.28 (0.23)	11.40 (SM)	384 (57)	8.92 (3.94)	ND

*Averages and standard deviations (in parentheses) represent three measurements. ^a^ND, not determined, BDL, below detection limit, SM, single measurement*.

From two clone libraries that were constructed using water samples from April and September 2009, two dominant phylotypes of bacterial *amoA* genes from the “uncultivated freshwater” cluster and *Nitrosospira* cluster 3 were detected (Table 4). Although the relative proportions of gene clones changed between these two samples, the dominant member of each cluster remained abundant in both gene clone libraries. Phylotypes from bacterial *amoA* gene clone libraries affiliating to the “uncultivated freshwater” cluster were dominant in zeolite biofilms developed from water sampled in January and May 2010, but *Nitrosospira* cluster 3 phylotypes were not found (Table 4). Phylotypes affiliating to an “uncultivated biofilm” cluster were also found in *amoA* gene clone libraries from zeolite biofilms, albeit at lower abundance than the “uncultivated freshwater” cluster, indicating enrichment of a different ammonia-oxidizer community on zeolite particles than those found within native water samples from April or September 2009.

**Table 4:** **Dominant AOB groups in native water samples and attached to zeolite particles as determined by clone libraries and sequencing of PCR-amplified *amoA* genes**.

Sequence affiliation^a^	Identity	Representation in clone libraries^b^			
		April 09 (water)	September 09 (water)	January 10 (zeolite)	May 10 (zeolite)
Uncultivated freshwater cluster^c^	482/492	19/50	36/50	28/43	11/32
Uncultivated freshwater cluster (GU121148)	483/489	ND	ND	8/43	18/32
Uncultivated freshwater cluster^d^	479/491	6/50	ND	ND	ND
*Nitrosospira* cluster 3 (JF936098)	483/489	19/50	12/50	ND	ND
*Nitrosospira* cluster 3 (JF936661)	474/489	6/50	2/50	ND	ND
Uncultivated biofilm cluster (FR773951)	480/483	ND	ND	7/43	3/32

*^a^Determined by closest BLAST relatives to uncultivated ammonia-oxidizing bacteria and by inference to phylogenetic tree created with representative sequences in ClustalW*.

*^b^Number of clones out of total number examined in library*.

*^c^Kim et al. (2008)*.

*^d^Chen et al. (2009)*.

*ND, not detected*.

### Effect of zeolite biofilm on nitrification activity at cold temperatures

**Figure 3:**
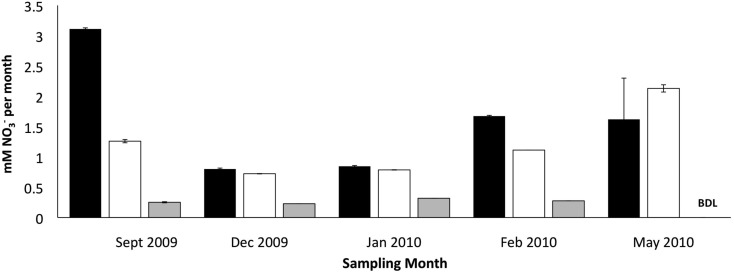
**Effect of zeolite biofilm on PNA measured as mM ${\text{NO}}_{\text{3}}^{-}$ produced per month in retention pond samples collected on indicated months**. Incubation conditions were 12°C with no zeolite addition (dark bars), 4°C with developed zeolite biofilm (1 g·5 L^−1^; white bars), and 4°C with no zeolite addition (gray bars). Error bars represent standard deviation of duplicate measurements for each sample. Samples were collected from sites 1 and 3 (Figure 1), and rate measurements were combined to calculate final rates and standard deviations.

**Figure 4:**
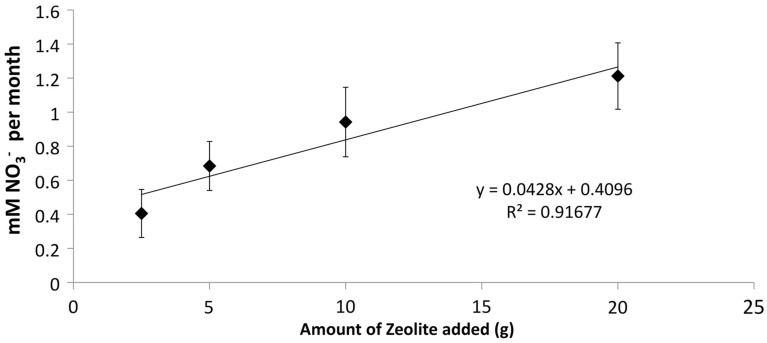
**Effect of increasing amounts of developed zeolite biofilm on PNA of retention pond samples collected from sites 1 and 3 (Figure 1) in January and May 2010**. Samples were incubated at 4°C. Rates and standard deviations were determined from duplicate measurements of the four samples (*n* = 8) over time.

**Figure 5:**
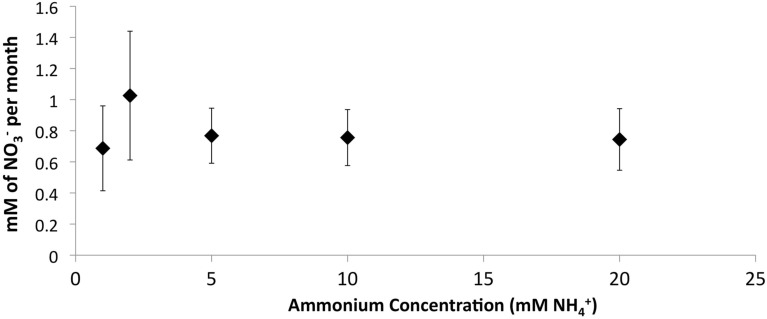
**Effect of increasing ${\text{NH}}_{\text{4}}^{\text{ + }}$ concentrations on PNA in retention pond samples collected from sites 1 and 3 (Figure 1) in May 2010 and containing developed zeolite biofilm (0.1 g mL^−1^)**. Samples were incubated at 4°C. Rates and standard deviations were determined from duplicate measurements of the two samples (*n* = 4) over time.

The addition of developed zeolite biofilm to retention pond water samples significantly increased the PNA at 4°C, measured as the rate of ${\text{NH}}_{\text{4}}^{\text{ + }}$ conversion to ${\text{NO}}_{\text{3}}^{-}$ (Figure 3). In the absence of zeolite biofilm, the rate of ${\text{NH}}_{\text{4}}^{\text{ + }}$ conversion to ${\text{NO}}_{\text{3}}^{-}$ at 4°C was 0–38% of rates measured at 12°C, whereas the presence of biofilm increased the rates to 41–131% at 4°C relative to those measured at 12°C (Figure 3). The amount of zeolite biofilm added to the water samples directly correlated with rates of PNA (Figure 4). The addition of increasing amounts of sterile zeolite had no effect on PNA, which remained statistically similar to rates found in water samples without any zeolite amendment (data not shown). This result indicates that the nitrifying microbial communities associated with zeolite were required at the beginning of the incubations in order to increase the measured rates of ${\text{NH}}_{\text{4}}^{\text{ + }}$ conversion to ${\text{NO}}_{\text{3}}^{-}$. Increasing concentrations of NH_4_Cl (1–20 mM) to water samples incubated with 0.1 g zeolite biofilm mL^−1^ water at 4°C had no effect on PNA, indicating that the nitrifying communities were saturated with substrate at 1 mM ${\text{NH}}_{\text{4}}^{\text{ + }}$, yet were not inhibited at up to 20 mM ${\text{NH}}_{\text{4}}^{\text{ + }}$ (Figure 5). Together, the data suggest that active nitrifying biomass, and perhaps the capacity of zeolite to bind ${\text{NH}}_{\text{4}}^{\text{ + }}$, accelerated conversion of ${\text{NH}}_{\text{4}}^{\text{ + }}$ to ${\text{NO}}_{\text{3}}^{\text{ - }}$ at 4°C and that this activity was not influenced by ${\text{NH}}_{\text{4}}^{\text{ + }}$ concentration nor the presence of zeolite itself without an associated nitrifying biofilm.

## Discussion

The primary finding of this study was that addition of zeolite to retention pond water from DDMI was capable of enriching and retaining an active nitrifying biomass, that when added to fresh water samples was capable of promoting ${\text{NH}}_{\text{4}}^{\text{ + }}$ removal to ${\text{NO}}_{\text{3}}^{-}$ at low temperature. The numbers of bacterial cells mL^−1^ in the native retention pond water were ca. 10-fold higher than those reported for natural ice-covered polar and alpine lakes (Alfreider et al., 1996; Garneau et al., 2008), which may be a consequence of its artificiality, designed specifically for remediation of mine leachate. As in many other cold aquatic systems, SSU rRNA genes of microorganisms from all three domains of life were detectable in the retention pond (Figure 2). The microbial community composition was more similar over the winter than in the summer months, when a phytoplankton bloom was evident, and many of the dominant DGGE bands recurred from May 2009 to May 2010. In addition, abundances of bacterial populations tended to shift from month to month (Table 2). Seasonal variability of microbial community composition has been observed in other aquatic ecosystems with seasonal ice cover (Pernthaler et al., 1998).

Gene markers of AOA have been found in high arctic lakes (Pouliot et al., 2009); however, the retention pond at DDMI was nearly devoid of detectable archaeal *amoA* genes and instead was dominated by two groups of bacterial *amoA* phylotypes (Tables 2 and 4). The abundance of *amoA* gene markers was not correlated to laboratory measurements of PNA of retention pond water, and tended to shift unpredictably (Table 2). Since the PNA measurements were conducted under optimal laboratory conditions with known quantities of ${\text{NH}}_{\text{4}}^{\text{ + }}$, variable *in situ* factors that dramatically influence microbial populations and activities were not accounted for. However, positive correlation of bacterial *amoA* gene abundance to pH and DO and negative correlation of PNA to ${\text{NH}}_{\text{4}}^{\text{ + }}$ concentration indicate that these particular *in situ* characteristics are reasonable indicators for presence and activity of nitrifying populations in this ecosystem, even though *amoA* gene abundance and PNA did not correlate directly to one another.

Development of nitrifying biofilms on zeolite particles enriched different ammonia-oxidizing microbial communities than those found in native water samples (Table 4); however, *amoA* gene clone libraries from all samples had high representation of phylotypes related to the “uncultivated freshwater” cluster. The difference in *amoA* phylotypes between water and biofilm samples could have reflected variation in the microbial community from month to month, but was more likely due to differential ability of particular ammonia-oxidizing taxa to attach to and grow on zeolite minerals. There are no published molecular studies of ammonia-oxidizing microbial communities attached to zeolite, although organisms belonging to the genus *Nitrosomonas* were previously identified in zeolite biofilms utilizing plate count methods (Pak et al., 2002; Chang et al., 2011).

The positive correlation between the amount of zeolite biofilm to rates of PNA (Figure 4) and the lack of response to sterile zeolite (data not shown) or increasing ${\text{NH}}_{\text{4}}^{\text{ + }}$ concentration (Figure 5) indicate that active biomass, perhaps bolstered by cation exchange properties of zeolite, is the primary limiting factor to nitrification at low temperature. In this study, laboratory measurements of PNA were appropriate for determining the effectiveness of zeolite in maximizing ${\text{NH}}_{\text{4}}^{\text{ + }}$ removal at low temperature, although these measurements did not show a predictable pattern reflective of *in situ* variations from month to month (Figure 3).

Altogether, the results suggest that addition of zeolite to the retention pond at DDMI could provide a cost-effective approach to maintain high rates of ${\text{NH}}_{\text{4}}^{\text{ + }}$ removal year-round by promoting the formation and retention of an active nitrifying biomass. The development of an active nitrifying biofilm on zeolite took 1 month in this study, suggesting that addition of zeolite to the retention pond over months when temperatures are at their highest would allow colonization by the relatively high population of AOB in the system (Tables 2 and 3). The concentrated and active biomass could then support continued ${\text{NH}}_{\text{4}}^{\text{ + }}$ removal during months of ice cover when water temperatures decrease (Table 3).

Application of zeolite to the retention pond could be accomplished by placing the mineral in removable mesh bags that could rest on the sediment surface. In warm months, the nitrifying biomass would colonize the zeolite and remain in the unfrozen part of the retention pond for maximum ${\text{NH}}_{\text{4}}^{\text{ + }}$ removal under ice cover. Although zeolite has been shown to sustain nitrifying biomass over several seasons in a wetland ecosystem (Gorra et al., 2007), emplacement of the mineral in removable bags would allow mining operators to control zeolite amount and application to suit their particular needs. The present study provides a proof-of-principle that zeolite can be utilized in environments such as mines, wastewater treatment facilities (Lahav and Green, 1998, 2000; Pak et al., 2002), and other N-impacted ecosystems to enhance nitrification and remediate nitrogenous wastes at low temperature, thus preventing ${\text{NH}}_{\text{4}}^{\text{ + }}$ accumulation during cold winter months or under seasonal ice cover.

## Conflict of Interest Statement

The authors declare that the research was conducted in the absence of any commercial or financial relationships that could be construed as a potential conflict of interest.
